# Multilocus sequence analysis of *Treponema denticola* strains of diverse origin

**DOI:** 10.1186/1471-2180-13-24

**Published:** 2013-02-04

**Authors:** Sisu Mo, Meng You, Yvonne CF Su, Donnabella C Lacap-Bugler, Yong-biao Huo, Gavin JD Smith, W Keung Leung, Rory M Watt

**Affiliations:** 1 Oral Biosciences, Faculty of Dentistry, The University of Hong Kong, Prince Philip Dental Hospital, 34 Hospital Road, Sai Ying Pun, Hong Kong; 2 Oral Diagnosis and Polyclinics, Faculty of Dentistry, The University of Hong Kong, Prince Philip Dental Hospital, 34 Hospital Road, Sai Ying Pun, Hong Kong; 3 Program in Emerging Infectious Diseases, Duke-NUS Graduate Medical School Singapore, 8 College Road, Singapore, 169857, Singapore

**Keywords:** *Treponema denticola*, Periodontal disease, Phylogeny, Multilocus sequence analysis, MLSA, Spirochete, Oral microbiota, Infectious diseases, Dentistry

## Abstract

**Background:**

The oral spirochete bacterium *Treponema denticola* is associated with both the incidence and severity of periodontal disease. Although the biological or phenotypic properties of a significant number of *T*. *denticola* isolates have been reported in the literature, their genetic diversity or phylogeny has never been systematically investigated. Here, we describe a multilocus sequence analysis (MLSA) of 20 of the most highly studied reference strains and clinical isolates of *T*. *denticola*; which were originally isolated from subgingival plaque samples taken from subjects from China, Japan, the Netherlands, Canada and the USA.

**Results:**

The sequences of the 16S ribosomal RNA gene, and 7 conserved protein-encoding genes (*flaA*, *recA*, *pyrH*, *ppnK*, *dnaN*, *era* and *radC*) were successfully determined for each strain. Sequence data was analyzed using a variety of bioinformatic and phylogenetic software tools. We found no evidence of positive selection or DNA recombination within the protein-encoding genes, where levels of intraspecific sequence polymorphism varied from 18.8% (*flaA*) to 8.9% (*dnaN*). Phylogenetic analysis of the concatenated protein-encoding gene sequence data (ca. 6,513 nucleotides for each strain) using Bayesian and maximum likelihood approaches indicated that the *T*. *denticola* strains were monophyletic, and formed 6 well-defined clades. All analyzed *T*. *denticola* strains appeared to have a genetic origin distinct from that of ‘*Treponema vincentii*’ or *Treponema pallidum*. No specific geographical relationships could be established; but several strains isolated from different continents appear to be closely related at the genetic level.

**Conclusions:**

Our analyses indicate that previous biological and biophysical investigations have predominantly focused on a subset of *T*. *denticola* strains with a relatively narrow range of genetic diversity. Our methodology and results establish a genetic framework for the discrimination and phylogenetic analysis of *T*. *denticola* isolates, which will greatly assist future biological and epidemiological investigations involving this putative ‘periodontopathogen’.

## Background

Periodontal disease is a chronic inflammatory infection that affects the tissues surrounding and supporting teeth [1-3]. It is highly prevalent in adult populations around the world, and is the primary cause of tooth loss after the age of 35 [2-4]. The term ‘periodontal disease’ encompasses a spectrum of related clinical conditions ranging from the relatively mild gingivitis (gum inflammation) to chronic and aggressive forms of periodontitis; where inflammation is accompanied by the progressive destruction of the gingival epithelial and connective tissues, and the resorption of the underlying alveolar bone. It has a highly complex, multispecies microbial etiology; typified by elevated populations of proteolytic and anaerobic bacterial species [5]. Oral spirochete bacteria, all of which belong to the genus *Treponema*, have long been implicated in the pathogenesis of periodontitis and other periodontal diseases [6]. One species in particular: *Treponema denticola* has been consistently associated with both the incidence and severity of periodontal disease [6-11].

Over the past few decades, a significant number of *T*. *denticola* strains have been isolated from periodontal sites in patients suffering from periodontal disease; predominantly from deep ‘periodontal pockets’ of infection that surround the roots of affected teeth. Clinical isolates of *T*. *denticola* have previously been identified and differentiated by a combination of cell morphological features; biochemical activities (e.g. proteolytic substrate preferences), immunogenic properties (e.g. serotyping, or reactivity towards monoclonal or polyclonal antibodies) as well as multilocus enzyme electrophoresis [12-17]. However, these approaches are generally tedious and technically demanding, and often yield inconsistent or ambiguous results.

To date, only two complete genome sequences are available for oral spirochete bacteria; those of *T*. *denticola* ATCC 35405 (type strain) [18] and *Treponema vincentii* LA-1 (ATCC 35580), which has been sequenced by researchers at the J. Craig Venter Institute as part of the Human Microbiome Project [19], but is as yet unpublished. The 2.84 Mbp single circular chromosome of *T*. *denticola* ATCC 35405 contains ca. 2,770 predicted protein-encoding genes, whilst the 2.51 Mbp *T*. *vincentii* genome is predicted to have ca. 2,600 protein encoding genes (NCBI GenBank accession number NZ_ACYH00000000). The syphilis spirochete *Treponema pallidum* is closely-related to *T*. *denticola* at the genetic level, but contains a much smaller ‘host-adapted’ genome ca. 1.14 Mbp in size [20].

Over recent years, multilocus sequence analysis (MLSA) has proven to be a powerful method for the discrimination, taxonomic classification and phylogenetic analysis of closely related microbial species, subspecies and strains [21-29]. MLSA involves the systematic comparison of the DNA sequences of sets of (conserved) genes, usually 2 to 10 in number, within a given set of strains or species. Commonly, the total gene sequence data for a single isolate is concatenated prior to analysis using a variety of distance-based or criterion-based computational methods. MLSA offers many advantages over ‘single gene’ approaches; most notably its greater sensitivity and resolving power, and its ability to identify or overcome conflicting signals, such as those arising from horizontal gene transfer [22,23,29].

Although studies have consistently associated *T*. *denticola* with periodontal disease, its precise pathogenic roles remain to be fully established. This issue has been complicated by the use of a variety of different *T*. *denticola* strains in previously reported biophysical analyses, cell culture-based investigations or animal infection models. Very little is presently known about how similar or disparate these isolates may be at the genetic level. This prompted us to utilize an MLSA-approach to systematically analyze the genetic composition of 20 of the most commonly used strains of *T*. *denticola*; originally isolated from patients with periodontal diseases who were living in Asia, Europe or North America. Our results reveal that there is considerable genetic diversity within this species. Phylogenetic analyses of multi-gene datasets indicate that the *T*. *denticola* strains studied share a common genetic origin, which is distinct from that of *T*. *vincentii* or *T*. *pallidum* and appear to have a clonal structure.

## Results

### Selection of strains and genetic loci for sequence analysis

All six ATCC reference strains of *T*. *denticola*, as well as 14 other clinical isolates were selected for multilocus sequence analysis (see Table 1). These strains were originally isolated from the oral cavities of subjects with various forms of periodontal disease; who resided in China, Japan, the Netherlands, Canada or the USA. We subjectively chose these particular strains based on several main criteria: 1) their diverse geographical origin; 2) their inclusion in one or more previously-published scientific investigations; and 3) their reported differences in phenotypic properties. Using the genome sequence of the type strain (ATCC 35405), seven protein-encoding genes distributed throughout the single, circular chromosome were selected for genetic analysis: *flaA*, *recA*, *pyrH*, *ppnK*, *dnaN*, *era* and *radC* (see Table 2). This approach enabled us to obtain a representative snapshot of genomic composition within each strain. None of these genes are predicted to reside in regions of suspected prophage origin [18]. Using a PCR-based strategy, the full length gene sequences for all seven genes were determined for each of the 19 other *T*. *denticola* strains. Details are shown in Table 3. Only the *era* gene from the ATCC 700768 strain could not be PCR-amplified using any primer set, and its sequence was determined by direct sequencing of purified chromosomal DNA. The gene sequences corresponding to the major rRNA component of the small ribosomal subunit (*rrs*, 16S rRNA) were also determined for each strain, to confirm their taxonomic assignment. In *T*. *denticola*, 16S rRNA is encoded by two genes (*rrsA*, *rrsB*), which have identical sequences and are positioned at distinct chromosomal loci (see Table 2) [18].

**Table 1:** **Origins of the *Treponema denticola *strains used in this study**

**Strain**	**Origin**	**Disease /isolation site(depositor)**	**Reference**
ATCC 35405^T^ (strain a)	Canada	Periodontal pocket (ECS Chan)	[30]
ATCC 35404 (strain c, TD-4)	Canada	Periodontal pocket (ECS Chan)	[30]
ATCC 33521 (strain 11)	USA	Subgingival plaque (RK Nauman)	[31]
ATCC 33520 (strain W)	USA	Subgingival plaque (RK Nauman)	[31]
GM-1	USA	Human periodontal pocket (SC Holt)	[32]
MS25	USA	Human periodontal pocket (SC Holt)	[32]
ST10	USA	(S. Socransky)	[33,34]
CD-1	USA	(WJ Loesche)	-
OTK	USA	(RC Johnson)	-
OT2B	USA	(RC Johnson)	-
NY535	Netherlands	Gingival biopsy of human periodontitis (FHM Mikx)	[35-37]
NY545	Netherlands	Gingival biopsy of human periodontitis (FHM Mikx)	[36,37]
NY531	Netherlands	Gingival biopsy of human periodontitis (FHM Mikx)	[36,37]
NY553	Netherlands	Gingival biopsy of human periodontitis (FHM Mikx)	[36,37]
ATCC 700771 (OMZ 834)	China	Chinese ANUG patient (C. Wyss)	[15]
ATCC 700768 (OMZ 830)	China	Chinese ANUG patient (C. Wyss)	[15]
OMZ 852	China	Chinese ANUG patient (C. Wyss)	[15]
OMZ 853	China	Chinese gingivitis patient (C. Wyss)	-
S2	Japan	(T. Eguchi)	[38]
OKA3	Japan	(T. Eguchi)	[38]

**Table 2:** **Gene regions used for multi locus sequence analysis of *Treponema denticola***

**Gene**	**Encoded protein/RNA**	Gene length* (bp)	Chromosomal Locus*
*dnaN* (*TDE0231*)	DNA polymerase III beta subunit	1104	264,000
*recA*(*TDE0872*)	Recombinase A	1245	895,000
*radC* (*TDE0973*)	Unknown function	678	998,000
*ppnK* (*TDE1591*)	Polyphosphate/ATP-NAD kinase	855	1,639,000
*flaA* (*TDE1712*)	Flagellar sheath protein	1050	1,767,000
*era* (*TDE1895*)	GTP binding protein	885	1,915,000
*pyrH* (*TDE2085*)	Uridylate kinase	696	2,114,000
*rrsA* (*TDE*_*16SA*)	Small subunit 16S rRNA (identical to *rrsB*)	1515	661,000
*rrsB* (*TDE*_*16SB*)	Small subunit 16S rRNA (identical to *rrsA*)	1515	1,220,000

*Chromosomal loci and gene lengths are based on the type strain of *T*. *denticola* (ATCC 35405) [18].

**Table 3:** **List of primers used for PCR amplification of protein**-**encoding genes from *Treponema denticola *strains**

**Gene**	**Primer**	**Sequence(5**^**′**^**to 3**^**′**^**)**	**Strains amplified**
*dnaN*	dnaNF	ATGAAAATAAGTTTTGACAGAGACAC	dnaF + dnaR: all strains (55-50°C)
	dnaNR	TTACTCCGTCTGCATAGGC	
*recA*	recAF1	GTGGCAAAAGCAAAAAAC	recAF1 + recAR1: most strains (55-47°C)
	recAR1	TTAAAAAAGACTGTCGTCCG	recAF2 + recAR2: ATCC 700768, MS25 (54-47°C)
	recAF2	TTCATATTGGCCGCATTTG	recAF1 + recArecAR2: ATCC 700771 (55-49°C)
	recAR2	TTGTGTACTCATAATGCCGCTC	
	recAF	GTGGCAAAAGCAAAAAACGAAG	recAF + recAR: OMZ852, OMZ853, NY531, NY553 (58-53°C)
	recAR	TTAAAAAAGACTGTCGTCCGCC	
*radC*	radCF1	ATGATAGACTATAAAAATTCGTCCAATAC	radCF1 + radCR1: most strains (55-50°C)
	radCR1	TTAAATATCAAACCTCGTTCCG	radCF1 + radCR2: MS25 (55-49°C)
	radCF2	AACATGGCTTTCCGAAATC	radCF2 + radCR1: ATCC 700768 (55-49°C)
	radCR2	GTGCAGCGGCTCTAAAAG	
*ppnK*	TDE1591F1	ATATGGATCCCATATGAAAAAAG	TDE1591F1 + TDE1591R1: most strains (52-45°C)
	TDE1591R1	AATTCTCGAGTCAATTCAGTTTGGG	TDE1591F2 + TDE1591R2: OKA3, MS25,GM1, ST10A,
	TDE1591F2	AGCTACCCTGCCCTAATTTC	ATCC 700768, ATCC 700771 (57-52°C)
	TDE1591R2	AACATCCTTAAAAAGCGGC	
*flaA*	TDE1712F	ATGAAAAAAACATTTATACTTGTTG	TDE1712F + TDE1712R: all strains (52-46°C)
	TDE1712R	TTATTGTTGGTTCTTTTCGG	
*era*	eraF1	ATGAACAGCGGAGTTGTAAC	eraF1 + eraR1: most strains (55-50°C)
	eraR1	TTAATACGAGATTTTTTTTATGATATTATC	
	eraF2	GGTACTTGTGCTTACCGAAAAC	eraF2 + eraR2: MS25 (54-47°C)
	eraR2	CCGACACAATCGAGGAAG	
	eraF4	CGCTTAGAAGAAGGGGATGC	eraF4, eraR4 separately used for direct chromosome sequencing of ATCC 700768^†^
	eraR4	CTTTTTCGACATAGAGGAAGGC	
*pyrH*	pyrHF	ATGGTAACTGTTTTGTCGGT	pyrHF + pyrHR: all strains (54-47°C)
	pyrHR	TTAGCCGATTACCGTTCCTT	

Genetic loci are based on the ATCC 35405 type strain of *Treponema denticola*.

F: Forward primer; R: Reverse primer. Values in parenthesis indicate annealing temperatures used in ‘touchdown PCR’ procedures.

^†^PCR amplification was unsuccessful; sequencing of chromosomal DNA employed.

### Inter-strain differences in nucleotide composition

We first compared the G + C content of each of the eight genes within the 20 *T*. *denticola* strains, to evaluate inter-gene and inter-strain variation. Results are summarized in Table 4. For all gene sequences, average G + C content (%) ranged from 32.4% to 52.4%. The *rrsA*/*B* gene had the highest average G + C content (52.4%), whilst the *dnaN* gene had the lowest (32.4%). The other six genes had similar overall levels of G + C content; ca. 40 − 45%. The G + C levels for individual genes exhibited very little variation between the strains (≤ ± 0.5%). Average overall G + C content for the eight genes in all 20 strains was ca. 42.5% (Additional file 1), which is slightly higher than the overall G + C content for the entire *T*. *denticola* ATCC 35405 genome, which is ca. 37.9% [18].

**Table 4:** **Summary of G** + **C content** (%), **number of polymorphic sites**, **nucleotide diversity per site**, **global rate ratios and the number of negatively selected codon sites for each gene selected for MLSA**

**Gene**	**No**. **of nucleotide sites**	**G +** **C (%)**	**No. (%)of polymorphic sites**	**Nucleotide diversity(Pi)**	**Global rate ω(95%CI)**	**No**. **of negatively selected sites**
*flaA*	1050	40.7 ± 0.4	197 (18.8)	0.0308 ± 0.0130	0.106 (0.080-0.132)	3
*recA*	1245	45.7 ± 0.5	147 (11.8)	0.0333 ± 0.0049	0.088 (0.065-0.111)	37
*pyrH*	696	41.8 ± 0.4	128 (18.4)	0.0331 ± 0.0125	0.064 (0.043-0.087)	11
*ppnK*	855	40.9 ± 0.5	85 (9.9)	0.0309 ± 0.0026	0.082 (0.053-0.110)	20
*dnaN*	1104	32.4 ± 0.2	98 (8.9)	0.0261 ± 0.0023	0.016 (0.006-0.026)	25
*era*	885	42.4 ± 0.4	115 (13.0)	0.0309 ± 0.0044	0.096 (0.068-0.123)	31
*radC*	678	43.3 ± 0.2	76 (11.2)	0.0275 ± 0.0048	0.032 (0.015-0.050)	19
16S rRNA	1497	52.4 ± 0.1	16 (1.1)	0.0018 ± 0.0005	N/A*	N/A*

* N/A: not applicable. These analyses are for protein-encoding genes.

Multiple sequence alignments were separately constructed for the eight genes, using sequence data from each of the 20 *T*. *denticola* strains. The eight respective sets of gene sequences aligned well, and there were only minor inter-strain differences in gene lengths. The number of polymorphic sites differed considerably between the seven protein-encoding genes (see Table 4); being highest in the *flaA* (18.8%) and *pyrH* (18.4%) genes, and lowest in the *dnaN* gene (8.9%). The 16S rRNA (*rrsA*/*B*) genes had by far the lowest numbers of polymorphic sites (1.1%), indicating a strong conservation of sequence.

### Phylogenetic analyses of *T*. *denticola* strains using individual gene sequence data

Using data obtained from the NCBI GenBank, gene homologues from *T*. *vincentii* LA-1 (ATCC 35580) and *T*. *pallidum* SS14 were also included in our phylogenetic analyses for comparative purposes (see Additional file 2). Homologues of the *flaA*, *recA*, *pyrH*, *ppnK*, *dnaN*, *era* and *radC* genes are present in *T*. *vincentii* LA-1. The *flaA*, *recA*, *pyrH*, *ppnK*, *dnaN* and *era* genes; but not *radC*, are present in *T*. *pallidum* (e.g. subsp. *pallidum* SS14 strain [39]). We first determined the most appropriate nucleotide substitution models to use; for the analysis of the 8 individual gene datasets, as well as the combined multi-gene datasets from each strain (species). Accordingly, the optimal nucleotide-substitution models were identified using the Akaike Information Criterion (AIC), as described by Bos and Posada [40]. The results are summarized in Additional file 3. The optimal nucleotide substitution model for the *recA*, *radC*, *dnaN* and *era* genes was the GTR + I + G model; whilst the GTR + G model was optimal for the 16S rRNA and *flaA* genes; and the K80 + I + G and SYM + G models were optimal for the *ppnK* and *pyrH* gene datasets, respectively. The respective optimal models were used for the phylogenetic analyses of the eight individual gene datasets, whilst the GTR + I + G model was used for the analysis of the concatenated seven-gene dataset (described below).

**Figure 1:**
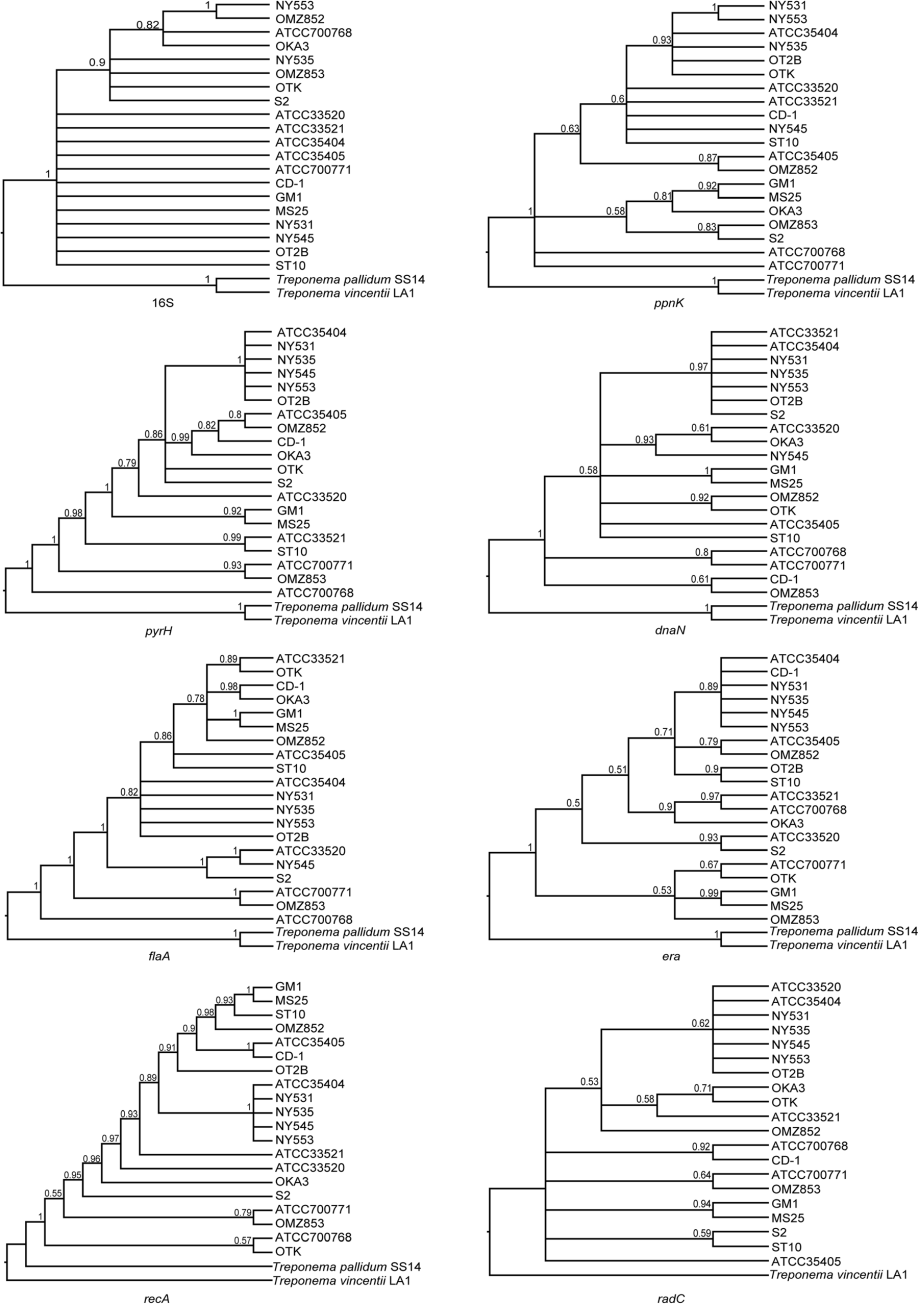
**Bayesian phylogenetic trees of *Treponema denticola *strains based on individual 16S rRNA, *flaA*, *recA*, *pyrH*, *ppnK*, *dnaN*, *era *and *radC *gene datasets.** The Bayesian 50% majority-rule consensus tree of 9,000 trees, following the removal of 1,000 trees as burn-in, is shown for each gene. Numbers above branches are posterior probabilities. Corresponding gene homologoues from *Treponema vincentii* LA-1 (ATCC 33580) and *Treponema pallidum* subsp. *pallidum* SS14 were included in the phylogenetic analysis as outgroups. The *radC* gene is absent from the *T*. *pallidum* genome.

Phylogenetic reconstructions based on the eight individual gene sequences (16S rRNA, *flaA*, *recA*, *pyrH*, *ppnK*, *dnaN*, *era* and *radC*) were performed using both maximum likelihood (ML) and Bayesian (BA) approaches. The eight BA trees constructed are shown in an ultrametric form (i.e. topology only) in Figure 1. The eight corresponding ML trees are shown with branch lengths proportional to genetic distances in Additional file 4. It should be noted that due to the proportionately large genetic distances between the *T*. *denticola*, *T*. *vincentii* and *T*. *pallidum* taxa, the two out-groups are not shown in the ML trees; so that the relationships between the respective *T*. *denticola* strains are more easily visualized (see below). Taken together, the 8 respective pairs of phylogenetic trees generated using these two different approaches shared similar overall topologies (i.e. had a similar shape and branching order). The 20 strains were fairly poorly resolved in the phylogenetic trees obtained from the individual 16S rRNA, *ppnK*, *radC* and *dnaN* gene datasets; especially in the ML trees; each forming polytomies (multifurcations) with a lack of statistical support. The BA topologies of the *flaA*, *recA*, and *pyrH* genes were the best resolved; especially on the backbone, indicating that 15 strains formed a well-supported monophyletic clade. However, the strain compositions and inter-strain relationships were not entirely concordant with one another. The MS25 and GM-1 strains formed a strongly supported clade in the *flaA*, *era*, *dnaN*, *recA* and *radC* trees generated by both phylogenetic approaches [BA: posterior probability (PP) = 0.99 − 1.00; ML: bootstrap support (BS) = 91 − 100]. The ATCC 35404, NY531, NY535 and NY553 strains clustered together in a strongly-supported clade in the *pyrH*, *dnaN* and *recA* trees constructed using both BA and ML methods.

**Figure 2:**
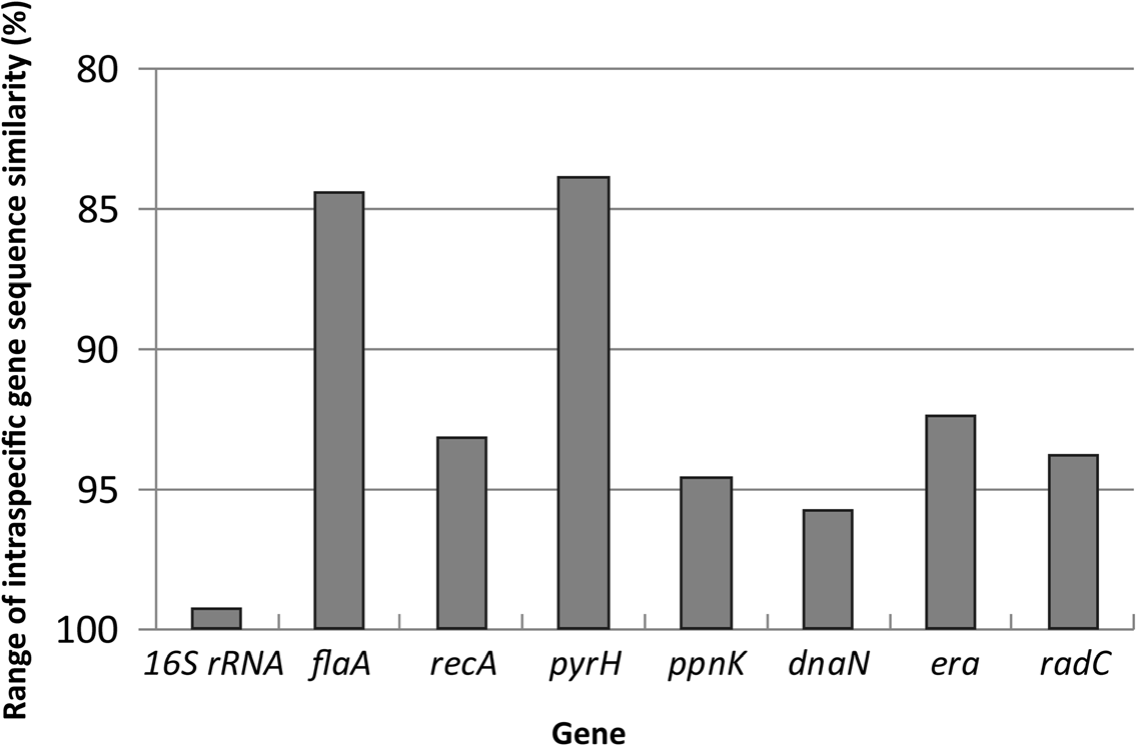
**Taxonomic resolution based on the ranges of intraspecific sequence similarity (%) for the individual 16S rRNA, *flaA***, ***recA***, ***pyrH***, ***ppnK***, ***dnaN***, ***era *and *radC *genes, within the 20 *Treponema denticola *strains analyzed.** The y-axis indicates the levels of nucleotide identity (%) shared between the eight individual gene sequences analyzed from each strain, with the range represented as a bar.

The range of intraspecific sequence similarity (%) was calculated for each gene, in order to determine how this measure of DNA sequence variation could be used to discriminate the 20 *T*. *denticola* strains (Figure 2). The *pyrH* gene had the highest levels of sequence polymorphism between the strains (83.9 − 100% similarity), closely followed by *flaA* (84.4 − 100%). The 16S rRNA gene had by far the lowest levels of inter-strain sequence variation (99.3 − 100% similarity). This indicated that the *pyrH* and *rrsA*/*B* gene sequences respectively had the best and worst strain-differentiating abilities. The levels of nucleotide diversity per site (Pi) within each of the eight genes are shown in Table 4. In the protein-encoding genes, Pi values ranged from ca. 0.033 (*pyrH*, *recA*) to 0.026 (*dnaN*).

### Detection of recombination using concatenated multi-gene sequence data

Failing to account for DNA homologous recombination (i.e. horizontal genetic exchange) can lead to erroneous phylogenetic reconstruction and also elevate the false-positive error rate in positive selection inference. Therefore, we checked for evidence of recombination within each of the eight individual genetic loci in all 20 strains, by identifying possible DNA ‘breakpoints’ using the HYPHY 2.0 software suite [41]. No evidence of genetic recombination was found within any gene sequences in any strain. This indicated that all the sites in the respective gene sequences shared a common evolutionary history.

### Analysis of selection pressure at each genetic locus

Selection pressure was analyzed by determining the ratios of non-synonymous to synonymous mutations (ω = *d*_N_/*d*_S_) for each codon site within each of the seven protein-encoding genes, in each of the 20 strains. When ω < 1, the codon is under negative selection pressure, i.e. purifying or stabilizing selection, to conserve the amino acid composition of the encoded protein. Table 4 summarizes the global rate ratios (ω = *d*_N_/*d*_S_) with 95% confidence intervals, as well as the numbers of negatively selected codon sites for each of the genes investigated. It may be seen that global ratios for the seven genes were subject to strong purifying selection (ω < 0.106), indicating that there was a strong selective pressure to conserve the function of the encoded proteins. No positively-selected sites were found in any of the 140 gene sequences.

### Phylogenetic analyses of *T*. *denticola* strains using concatenated multi-gene sequence data

**Figure 3:**
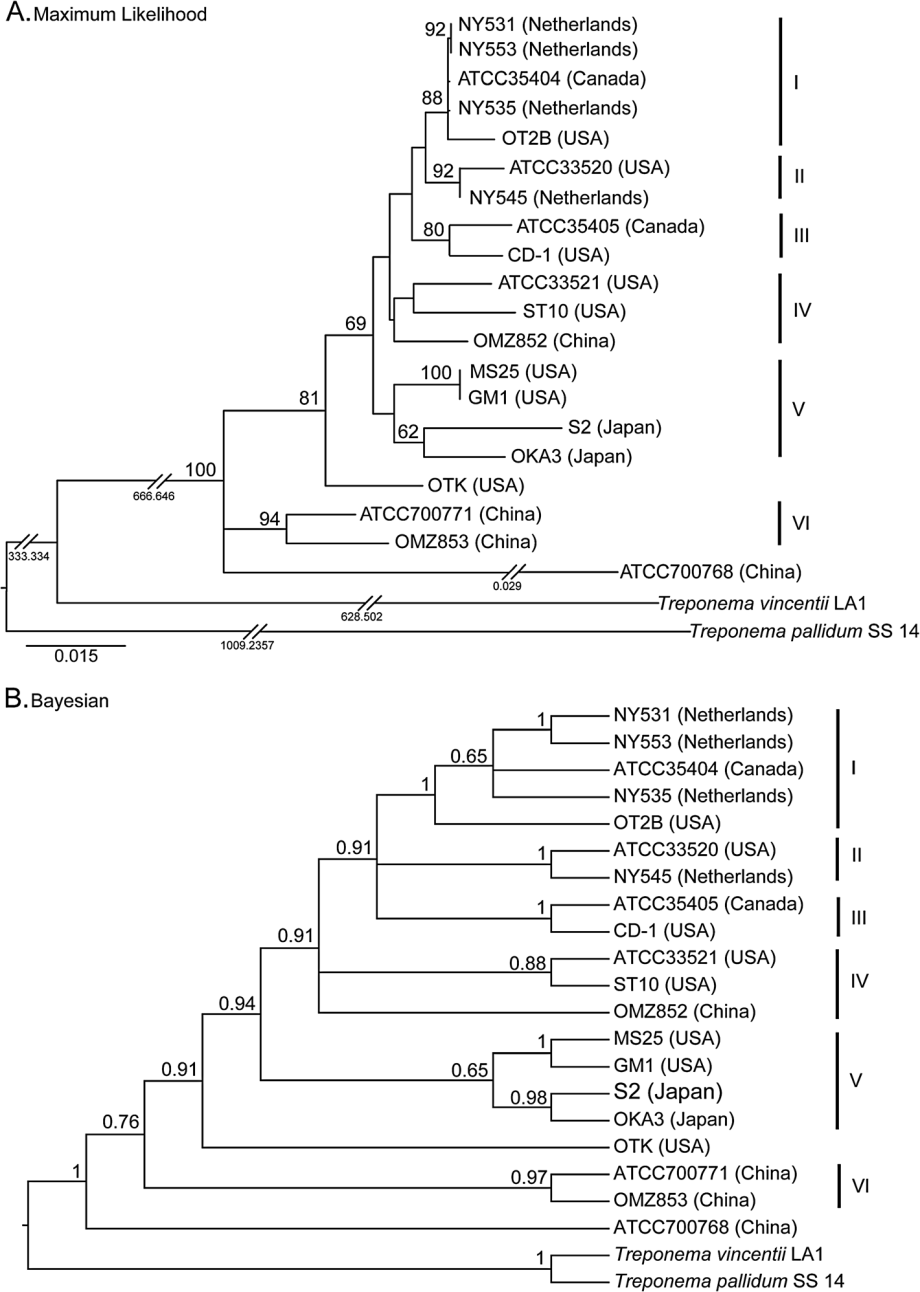
**Phylogenetic trees of *Treponema denticola *strains based on a concatenated 7**-**gene dataset** (***flaA***, ***recA***, ***pyrH***, ***ppnK***, ***dnaN***, ***era *and *radC***), **using Maximum Likelihood and Bayesian methods.A**: Maximum likelihood (ML) tree generated under the GTR + I + G substitution model, with bootstrap values shown above branches. The scale bar represents 0.015 nucleotide changes per site. Numbers beneath the breakpoints in the branches indicate the respective nucleotide changes per site that have been removed. **B**: Ultrametric Bayesian (BA) 50% majority-rule consensus tree of 9,000 trees following the removal of 1,000 trees as burn-in. Numbers above branches are posterior probabilities. The respective clades formed in each tree are indicated with a Roman numeral (I-VI). Corresponding gene homologoues from *Treponema vincentii* LA-1 (ATCC 33580) and *Treponema pallidum* subsp. *pallidum* SS14 were included in the phylogenetic analysis as outgroups.

The DNA sequences of the seven protein-encoding genes were concatenated in the order: *flaA* − *recA* − *pyrH* − *ppnK* − *dnaN* − *era* − *radC*, for analysis using BA and ML approaches. The combined data matrix contained 6,513 nucleotides for each strain. The ML tree is shown with branch lengths proportional to genetic distances (Figure 3A), whilst the BA tree is shown in an ultrametric form (Figure 3B). Both the BA and ML trees clearly show that the *T*. *denticola* strains share a monophyletic origin. The genetic distances on the ML tree indicate that the *T*. *denticola* strains analyzed here are much more closely related to each other, than to *T*. *vincentii* or *T*. *pallidum*. Six analogous clades (labeled I–VI) comprising 18 strains were identified in both the ML and BA trees. Clade I consists of five strains: NY531, NY553, ATCC 35404, NY535 and OT2B; with moderate to strong statistical support (BA PP = 1.00, ML BS = 88). Clade II has two strains (ATCC 33520 and NY545) and is well-supported (BA PP = 1.00; ML BS = 92). Clade III contains the CD-1 and ATCC 35405 (type) strains, which are both North American in origin, with moderate to strong support (BA PP = 1.00; ML BS = 80). Clade IV contains 3 strains (ATCC 33521, ST10 and OMZ 852) with no statistical support. Clade V comprises four strains: MS25, GM-1, S2 and OKA3. Although this clade has no support, it is apparent that the two USA strains (MS25 and GM-1) form a well-supported clade (BA PP = 1.00, ML BS = 100), whereas the two Japanese strains (S2 and OKA3) form a clade with moderate to strong support (BA PP = 0.98, ML BS = 62). Clade VI comprises two strains from China (ATCC 700771 and OMZ 853), with strong support (BA PP = 0.97, ML BS = 94). The Chinese ATCC 700768 strain is found to be basal to the other 19 strains in the BA tree, and appears to be highly divergent in the ML tree. Since the ML tree is better resolved than the corresponding BA tree, we will primarily refer to the ML tree in the rest of this paper.

## Discussion

The oral spirochete bacterium *Treponema denticola* is postulated to play an important role in the pathogenesis of periodontal disease; in particular chronic periodontitis, which is estimated to affect ca. 10-15% of the global population [3,4,6-9]. It is also implicated in the etiology of acute necrotizing ulcerative gingivitis (ANUG) [42] and orofacial noma [43], two other tissue-destructive diseases of the orofacial region. However, *T*. *denticola* is commonly detected in the oral microbiota in dentulous adults; albeit at relatively low levels, and its precise etiopathogenic mechanisms remain to be established. The elucidation of more specific disease associations is presently hampered by the lack of a reliable method for strain identification, and a very poor understanding of how strains differ at the genetic level.

Here, we utilized a seven protein-coding gene multilocus sequence analysis (MLSA) approach, to characterize genomic diversity and evolutionary relationships in a small, but carefully-selected collection of *T*. *denticola* isolates of diverse geographical origin. Our results revealed that there are relatively high levels of genetic diversity amongst *T*. *denticola* strains; with gene sequence similarities ranging between ca. 84 − 100% between the strains. These levels are considerably higher than in *T*. *pallidum*; where strains of the *pallidum* and *pertenue* subspecies share ca. 100-99.6% genome sequence identity, and genetic differences are largely confined to recombination ‘hotspots’ or other areas of acquired DNA sequence [20]. Whilst there were variations in the relative proportions of polymorphic sites present in the seven protein-encoding genes selected for analysis, all were under a strong (purifying) evolutionary pressure to conserve function. We found no evidence of genetic recombination in any gene sequence analyzed, indicating that genes had evolved as intact units in each strain. It is interesting to note that the *flaA* gene, which encodes an endoflagellar sheath protein that is a known a cell surface-exposed epitope [44], appeared to follow a similar evolutionary pathway as the *pyrH* and *recA* ‘housekeeping’ genes analyzed. Although we also sequenced the 16S rRNA (*rrsA*/*rrsB*) gene(s) from each strain, we did not add this to the concatenated multi-gene sequence for phylogenetic analysis. This was because it is present in two identical copies on the *T*. *denticola* genome [18], and may be under distinct evolutionary pressures, due to the fact that is not translated into a protein; e.g. it may have increased levels of nucleotide insertions or deletions (indels), or may have selection biases relating to its secondary structure [24].

Based on the concatenated 7-gene (*flaA*, *recA*, *pyrH*, *ppnK*, *dnaN*, *era* and *radC*) datasets, both the Bayesian (BA) and maximum likelihood (ML) topologies clearly indicated that all 20 *T*. *denticola* strains are monophyletic; i.e. they originated from a single common ancestor that was genetically distinct from *T*. *vincentii* and *T*. *pallidum* (see Figure 3). Our data also indicates that at the genetic level, *T*. *denticola* is more closely related to the oral treponeme *T*. *vincentii*, than the syphilis spirochete. Six well-defined clades (I-VI) were formed in both the BA and ML trees, which comprised 18 of the 20 strains analyzed. The OTK strain from the USA does not fall within any of the defined clades, possibly due to the relatively low sample size. The early-branching ATCC 700768 strain from China appears to be highly divergent from the other *T*. *denticola* taxa (discussed further below). The overall concordances in tree topologies obtained for the 7 individual genes, which are well-distributed around the ca. 2.8 Mbp chromosome, are consistent with *T*. *denticola* being predominantly clonal in nature. We did not attempt to estimate evolutionary timescales, as the precise dates of isolation are not known for these strains. Due to the high levels of sequence divergence and putatively clonal strain distributions, we speculate that *T*. *denticola* has been co-evolving in humans and animal hosts for a considerable period of time. However, genome sequence data from additional strains of known isolation date will be required to validate this proposition.

It should be noted that the majority of previous biophysical or culture-based investigations involving *T*. *denticola* have primarily utilized only three different (ATCC) strains: 35405^T^ (Clade III), 35404 (Clade I) and 33520 (Clade II); which are all of North American origin [30,31]. Our data suggests that these three strains (lineages) may not be wholly representative of the *T*. *denticola* strains distributed within global populations. Whilst our sample size is modest, the scope of our MLSA analysis was limited by the relative paucity of *T*. *denticola* strains presently available. Oral treponemes such as *T*. *denticola* are fastidious, capricious and notoriously difficult to isolate; and there are very few laboratories in the world that actively maintain strain collections.

The ATCC 700768 (OMZ 830, China), ATCC 700771 (OMZ 834, China), OMZ 853 (China) and OTK (USA) strains, located in basal positions in the phylogenetic trees, appear to be the most genetically distant from the genome-sequenced ATCC 35405 type strain (Canada). This genetic divergence is consistent with literature reports, which have stated that these strains have notable phenotypic differences. For example, the primary sequence, domain structure and immunogenic properties of the major surface protein (Msp) in the OTK strain, were shown to be quite distinct from those of the ATCC 35405 or 33520 strains [14,45,46]. In another study, Wyss *et al*. reported that the FlaA proteins from the ATCC 700768 and ATCC 700771 strains reacted positively towards the ‘pathogen-related oral spirochete’ (PROS) H9-2 antibody (raised against *T*. *pallidum*); whilst the ATCC 35405, 35404, 33521, 33520 and ST10 strains were unreactive [15].

It is highly notable that several sets of *T*. *denticola* strains with similar genetic compositions were isolated from subjects living on different continents; i.e. the MS25 (USA), GM-1 (USA), S2 (Japan) and OKA3 (Japan) strains in Clade V; the ATCC 33520 (USA) and NY545 (Netherlands) strains in Clade II; the ATCC 33521 (USA), ST10 (USA) and OMZ 852 (China) strains in Clade IV; and the ATCC 35404 (Canada), OT2B (USA), NY531 (Netherlands), NY535 (Netherlands) and NY553 (Netherlands) strains in Clade I. We tentatively propose that this indicates that there may be a number of *T*. *denticola* clonal lineages, or closely-related clusters of strains, which have global distributions. We also identified closely-related strains that had been isolated from different subjects residing in the same geographical location: e.g. the ATCC 700771 and OMZ 853 strains from China (Clade VI).

This study represents the first in-depth multilocus sequencing approach that has been used to analyze strains belonging to a species of oral spirochete bacteria. However, it is important to note that alternative MLSA schemes have previously been used to characterize intra-species variation in other (pathogenic) spirochetes. A 21 gene MLSA approach was notably used to probe the origins, evolutionary history and possible migratory routes of *T*. *pallidum*, the causative agent of syphilis [28]. Genetic diversity within *Borellia burgdorferi sensu lato*, was similarly investigated using a seven gene MLSA system [27], enabling taxonomic relationships to be defined within this complex group of related (sub)-species. As far as other putative periodontal pathogens are concerned, Koehler and coworkers used a 10 gene MLSA system to investigate genetic relationships between 18 *Porphyromonas gingivalis* strains isolated from patients with periodontitis in Germany, and one isolate from the USA [47]. This revealed the presence of high levels of horizonal gene transfer, i.e. a panmictic population structure; quite unlike what we have found for *T*. *denticola* here. Subsequent studies have revealed that both *P*. *gingivalis* and another another ‘periodontopathogen’: *Aggregatibacter actinomycetemcommitans* both had specific lineages with increased association with periodontal disease; with apparently differing levels of carriage in certain ethnic groups or geographical populations [48-50]. It remains to be established whether *T*. *denticola* also possesses lineages with increased association with periodontal disease.

As the seven selected genes appear to be well-conserved in treponeme species, we envisage our MLSA framework as being readily adaptable for strain typing, as well as establishing intra- and inter-species phylogenetic relationships within diverse treponeme populations. For example, one interesting application would be to explore similarities and evolutionary relationships between closely-related strains and species of treponeme bacteria found in the human oral cavity, versus those present in animal reservoirs; especially those associated with polymicrobial tissue-destructive infections [51,52].

## Conclusions

Our sequencing data clearly reveals that clinical isolates of the periodontal pathogen *T*. *denticola* have highly diverse genotypes. We define 6 distinct clonal lineages present within strains isolated from subjects living in Asia, Europe and North America. Several *T*. *denticola* lineages are present on different continents, which is consistent with the existence of strains with widespread, possibly global, distributions. Our results lay the foundations for future systematic molecular investigations aimed at establishing the ecological distributions, disease associations or phylogeny of treponemes belonging to this and other species.

## Methods

### Strain culture; gene amplification, cloning and sequencing

*Treponema denticola* strains were purchased from the American Type Culture Collection (ATCC) or generously provided by Dr. Barry McBride (University of British Columbia, Canada), Dr. Chris Wyss (University of Zurich, Switzerland) and Dr. E. Peter Greenberg (Washington University, USA). All strains were cultured anaerobically in TYGVS media supplemented with 10% rabbit serum as previously described [53]. Genomic DNA was purified from 3-5 day old cultures using a Wizard Genomic DNA Purification Kit (Promega), using the manufacturer’s gram-negative protocol. PCR primers targeting the *dnaN* (TDE0231); *recA* (TDE0872); *radC* (TDE0973); *ppnK* (TDE1591); *flaA* (TDE1712); *era* (TDE1895) and *pyrH* (TDE2085) genes were designed using Omiga 2.0 (Oxford Molecular), based on the genome-sequenced ATCC 35405 strain [18], and are listed in Table 3. The *rrsA*/*B* genes were amplified using the TPU1 (5^′^-AGAGTTTGATCMTGGCTCAG-3^′^) [54] and C90 (5^′^-GTTACGACTTCACCCTCCT-3^′^) primers [55]. PCR reactions were performed using a ‘touchdown’ method on a GeneAmp PCR System 9700 (Applied Biosystems). PCR reactions (50 μl) contained 10 μl of PyroBest Buffer II, 2 μl of genomic DNA (ca. 50 ng), 4 μl of dNTPs (2.5 mM each), 2 μl of each forward and reverse primer (10 μM each), and 0.25 μl of PyroBest DNA polymerase (1.25 U, TaKaRa). PCR cycling conditions consist of an initial denaturation (94°C, 90s); followed by 4-6 cycles of: denaturation (94°C, 20s), annealing (temperature as indicated in Table 3, 20s) decreasing 1°C every cycle, extension (72°C, 3 min); followed 26 cycles of denaturation (94°C, 15s), annealing (temperature as indicated, 15s), extension (72°C, 2 min); final extension (72°C, 7 min). PCR products were analyzed using 1% agarose gel electrophoresis and stained with ethidium bromide. PCR products were gel-purified using a QIAquick Gel Extraction Kit (Qiagen), and cloned into pCR2.1-TOPO vector using a TOPO TA Cloning Kit (Invitrogen) according to the manufacturer’s instructions. Ligation mixtures were electroporated into *Escherichia coli* DH10B cells, plated on Luria-Bertani (LB) 1% agar plates supplemented with kanamycin (50 μg/ml) and X-gal (5-bromo-4-chloro-indolyl-β-D-galactopyranoside, 20 μg/ml), and incubated overnight at 37°C. Plasmid DNA was purified from 4 or 5 colonies from each plate using the QIAprep Spin Miniprep Kit (Qiagen). At least three colonies containing PCR inserts were commercially sequenced in both directions (M13 forward and reverse primers) using an Applied Biosystems 3730xl DNA Analyzer. Direct sequencing of genomic DNA (Invitrogen, Hong Kong) using outward-facing internal primers was used as indicated to obtain genomic sequence data when PCR amplification proved unsuccessful.

### Analysis of gene sequence similarity and phylogeny

Sequence data were edited and assembled in Omiga 2.0 and EMBOSS GUI (European Molecular Biology Open Software Suite [56] and gene alignments were manually checked and optimized using BioEdit v.7.0.9 [57] and MEGA 4 [58]. GC content and the location of polymorphic sites were analyzed using Omiga 2.0 and FaBOX [59] (http://www.birc.au.dk/software/fabox). All seven genes (*flaA*, *recA*, *pyrH*, *ppnK*, *dnaN*, *era*, and *radC*) were concatenated using Se-Al ver.2.0a11 [60], giving a final alignment of 6,780 nucleotides (including gaps). The range of intraspecific sequence similarity (%) for each gene was calculated using the sequence identity matrix program implemented in BioEdit. Nucleotide polymorphism in each gene was evaluated by quantifying the nucleotide diversity per site (Pi) using DNA Sequence Polymorphism software (DnaSP 5.10) [61].

Maximum Likelihood (ML) and Bayesian methods were used to analyze both individual genes, and concatenated gene sequence datasets. The optimal substitution model and gamma rate heterogeneity for individual genes and combined dataset were determined using the Akaike Information Criterion (AIC) in MrModeltest ver. 2.2 [62]. Maximum likelihood (ML) trees were generated using GARLI ver. 0.96 [63] with support calculated from 100 bootstrap replicates. Bootstrap support (BS) values ≥ 70% were considered to have strong support.

Partitioned Bayesian analyses (BA) were conducted using MrBayes v.3.1.2 [64], with two independent runs of Metropolis-coupled Markov chain Monte Carlo (MCMCMC) analyses, each with 4 chains and 1 million generations, with trees sampled every 100 generations. The level of convergence was assessed by checking the average standard deviation of split frequencies (<0.005). Convergence of the runs was also checked visually in Tracer ver. 1.5 [65], ensuring the effective sample sizes (ESS) were all above 200. Bayesian posterior probabilities (PP) were calculated by generating a 50% majority-rule consensus tree from the remaining sampled trees after discarding the burn-in (10%). PP values ≥ 0.95 indicate statistical support.

### Detection of recombination and natural selection

A codon-based approach implemented in HYPHY 2.0 [41] was used to analyze selection pressures within the seven individual protein-encoding genes, using a neighbor-joining model. Genetic algorithm recombination detection (GARD) was first used to identify any possible recombination breakpoints within each gene. Single likelihood ancestor counting (SLAC) was employed to calculate the global nonsynonymous (*d*_N_) and synonymous (*d*_S_) nucleotide substitution rate ratios (ω = *d*_N_/*d*_S_), with 95% confidence intervals; and to test the selection of variable codon sites based on the most appropriate nucleotide substitution model and tree topology, with a critical p-value of 0.05.

### Nucleotide sequence accession numbers

Nucleotide sequences were submitted to GenBank under the following accession numbers: JF700256–JF700268 and KC415232−KC415235, [16S rRNA (*rrsA*/*B*)]; JF700269–JF700283 and KC415220−KC415223 (*flaA*); JF700284–JF700298 and KC415208−KC415211 (*recA*); JF700299–JF700313 and KC415216−KC415219 (*pyrH*); JF700314–JF700328 and KC415204−KC415207 (*ppnK*); JF700329–JF700343 and KC415228−KC415231 (*dnaN*); JF700344–JF700358 and KC415224−KC415227 (*era*); and JF700359–JF700373 and KC415212−KC415215 (*radC*). The *Treponema vincentii* LA-1 (ATCC 35580) and *Treponema pallidum subsp*. *pallidum SS14* reference strains were selected as outgroups, using complete genomes obtained from GenBank under Accession numbers NZ_ACYH00000000 and NC_010741, respectively.

## Abbreviations

MLSA: Multilocus sequence analysis; BA: Bayesian; ML: Maximum likelihood; PP: Posterior probability; BS: Bootstrap support; AIC: Akaike Information Criterion; RELL: Resampling of estimated log likelihoods; MCMCMC: Metropolis-coupled Markov chain Monte Carlo; GTR: General Time Reversible model; HKY: Hasegawa, Kishino and Yano model; SYM: Symmetrical model; I: Invariant sites; G: Gamma distribution of changes; dNTP: Deoxyribonucleic acid; rRNA: Ribosomal ribonucleic acid; ATCC: American type culture collection; TYGVS: Tryptone-yeast extract-gelatin-volatile fatty acids-serum (medium).

## Competing interests

The authors declare no competing interests; financial or otherwise.

## Authors’ contributions

Conceived the study: RMW. Designed and performed the practical experimental work: SM, MY, DCLB, YBH, WKL, RMW. Designed and performed the computational analyses: SM, MY, YCFS, DCLB, GJDS, RMW. Wrote the manuscript: SM, MY, YCFS, DCLB, GJDS, WKL, RMW. All authors have read and approved the final manuscript.

## Supplementary Material

Additional file 1 **Table summarizing G + C content (%) for the eight genes selected for sequence analysis within the 20 *Treponema denticola *strains.** Click here for file

Additional file 2 **Table summarizing details of the *flaA*, *recA*, *pyrH*, *ppnK*, *dnaN*, *era and radC *gene homologues present in *Treponema pallidum *SS14 and *Treponema vincentii *LA-1 (ATCC 35580).** Click here for file

Additional file 3 **Table summarizing the optimal models and parameter values for the individual gene and concatenated *flaA* − *recA* − *pyrH* − *ppnK* − *dnaN* − *era*** − ***radC *gene datasets analyzed in this study.** Click here for file

Additional file 4 **Maximum likelihood (ML) phylogenetic trees obtained for the individual 16S rRNA, *flaA*, *recA*, *pyrH*, *ppnK*, *dnaN*, *era *and *radC *gene datasets.** Click here for file
