# Supplementary material for: Multilocus sequence analysis of Treponema denticola strains of diverse origin
Source: BMC Microbiol. 2013 Feb 4;13:24. doi: 10.1186/1471-2180-13-24 (PMC3574001; doi:10.1186/1471-2180-13-24)
Supplement: Additional file 1 — Table summarizing G + C content (%) for the eight genes selected for sequence analysis within the 20 Treponema denticola strains. [file 1471-2180-13-24-S1.pdf]

Additional File 1. Table summarizing G+C content (%) for the eight genes selected for sequence analysis within the 20 *Treponema denticola* strains

Strain	16S rRNA	*flaA*	*recA*	*pyrH*	*ppnK*	*dnaN*	*era*	*radC*	% G+C
35405	52.4	41	46	42.2	40.8	32	42.3	43.5	42.5 ± 5.7
35404	52.4	41	46.1	41.8	40.8	32.3	42.6	43.1	42.5 ± 5.6
33520	52.4	41	46.1	42	41.6	32.9	42.3	43.1	42.7 ± 5.4
33521	52.4	40.9	45.9	41.4	41.3	32.3	41.6	43.2	42.4 ± 5.6
700768	52	39.8	44.6	40.4	40.4	32.8	42.3	43.4	42.0 ± 5.4
700771	52.5	40.2	45.2	42.2	40.8	32.3	42.7	43.5	42.4 ± 5.6
OTK	52.3	41	45.5	42	40.8	31.8	42.5	43.4	42.4 ± 5.7
OT2B	52.4	40.9	45.5	41.8	40.8	32.3	42	43.1	42.4 ± 5.6
MS25	52.4	40.7	45.8	41.8	40.8	32.5	41.9	43.1	42.4 ± 5.6
GM1	52.4	40.7	45.8	41.8	40.8	32.5	41.9	43.1	42.4 ± 5.6
NY535	52.4	41	46.1	41.8	40.8	32.3	42.6	43.1	42.5 ± 5.6
NY545	52.4	41	46.1	41.8	41.5	32.5	42.6	43.1	42.6 ± 5.5
OKA3	52.4	40.7	45.9	42	40.9	32.2	42.5	43.4	42.5 ± 5.6
S2	52.4	40.2	44.9	41.5	39.3	32.3	42.1	43.7	42.0 ± 5.7
CD1	52.4	40.6	45.9	42	41.5	32.5	42.6	42.9	42.6 ± 5.5
ST10	52.4	40.8	45.6	41.4	41.2	32.4	42.5	43.5	42.5 ± 5.7
NY531	52.4	40.9	46.1	41.8	40.8	32.3	42.6	43.1	42.5 ± 5.6
NY553	52.3	40.9	46.1	41.8	40.8	32.3	42.6	43.1	42.5 ± 5.6
OMZ852	52.3	40.7	45.8	42	41.1	32.3	41.5	43.4	42.4 ± 5.6
OMZ853	52.4	40.1	44.5	42.2	40.2	32.6	43.2	43.4	42.3 ± 5.5
**% G+C**	52.4 ± 0.1	40.7 ± 0.4	45.7 ± 0.5	41.8 ± 0.4	40.9 ± 0.5	32.4 ± 0.2	42.4 ± 0.4	43.3 ± 0.2	
