# Supplementary material for: Multilocus sequence analysis of Treponema denticola strains of diverse origin
Source: BMC Microbiol. 2013 Feb 4;13:24. doi: 10.1186/1471-2180-13-24 (PMC3574001; doi:10.1186/1471-2180-13-24)
Supplement: Additional file 2 — Table summarizing details of the flaA, recA, pyrH, ppnK, dnaN, era and radC gene homologues present in Treponema pallidum SS14 and Treponema vincentii LA-1 (ATCC 35580). [file 1471-2180-13-24-S2.pdf]

Additional File 2. Table summarizing details of the *flaA, recA, pyrH, ppnK, dnaN, era and radC* gene homologues present in *Treponema pallidum* SS14 and *Treponema vincentii* LA-1 (ATCC 35580)

**Gene homologue in *T. denticola ATCC 35405***	**Gene homologue in *T. pallidum* SS14 (locus tag), chromosomal loci and gene length**	**Gene homologue in *T. vincentii* LA1 (locus tag), chromosomal loci and gene length**
rrsA (TDE_16SA)	*rrs1* (TPASS_r0001), 231345, 1495bp	*rrs* (TREVI0001_2671), 150, 1518bp
rrsB (TDE_16SB)	*rrs2* (TPASS_r0004), 279779, 1495bp	-
dnaN (TDE0231)	*dnaN* (TPASS_0002), 1641, 1116bp	*dnaN* (TREVI0001_1741), 53288, 1104bp
recA (TDE0872)	*recA* (TPASS_0692), 760177, 1224bp	*recA* (TREVI0001_1268), 159410, 1326bp
radC (TDE0973)	-	*radC* (TREVI0001_1068), 17857, 669bp
ppnK (TDE1591)	*ppnK* (TPASS_0441), 468479, 918bp	*ppnK* (TREVI0001_1223), 109263, 852bp
flaA (TDE1712)	*flaA1* (TPASS_0249), 262628, 1053bp	*flaA* (TREVI0001_1368), 12473, 1062bp
era (TDE1895)	*era* (TPASS_0541), 583621, 960bp	*era* (TREVI0001_1582), 114822, 675bp
pyrH (TDE2085)	*pyrH* (TPASS_0099), 107370, 756bp	*pyrH* (TREVI0001_1144), 26015, 687bp

(-) Indicates gene homologue is absent
