# Supplementary material for: Multilocus sequence analysis of Treponema denticola strains of diverse origin
Source: BMC Microbiol. 2013 Feb 4;13:24. doi: 10.1186/1471-2180-13-24 (PMC3574001; doi:10.1186/1471-2180-13-24)
Supplement: Additional file 3 — Table summarizing the optimal models and parameter values for the individual gene and concatenated flaA − recA − pyrH − ppnK − dnaN − era − radC gene datasets analyzed in this study. [file 1471-2180-13-24-S3.pdf]

Additional File 3. Table summarizing the optimal models and parameter values for the individual gene and concatenated *flaA-recA-pyrH-ppnK-dnaN-era-radC* gene datasets analyzed in this study

Gene(s)	AIC selected model	Base frequencies				Substitution model (rate matrix)						I	G
		A	C	G	T	A-C	A-G	A-T	C-G	C-T	G-T		
16S rRNA	GTR+G	0.2357	0.2613	0.2666	0.2363	1.0570	8.9360	2.9364	1.0696	11.9956	1.0000	0.0000	0.6550
recA	GTR+I+G	0.2912	0.2228	0.2727	0.2132	1.5654	3.4724	0.7586	0.4473	7.7992	1.0000	0.3448	0.1719
pyrH	SYM+G	Equal frequencies	Equal frequencies	Equal frequencies	Equal frequencies	3.9326	4.1495	1.4383	0.5956	5.5201	1.0000	0.0000	0.3494
ppnK	K80+I+G	Equal frequencies	Equal frequencies	Equal frequencies	Equal frequencies	Ti/tv ratio= 2.7079						0.3299	0.2121
dnaN	GTR+I+G	0.3414	0.2078	0.1718	0.2790	0.9028	2.8849	0.3182	0.0460	9.4123	1.0000	0.3706	0.2305
era	GTR+I+G	0.2863	0.2750	0.2382	0.2005	1.7071	3.5460	0.4429	0.4104	5.4268	1.0000	0.3534	0.1931
radC	GTR+I+G	0.2782	0.2148	0.2520	0.2551	1.5893	3.4102	0.3895	0.6647	6.8748	1.0000	0.4475	0.2050
flaA	GTR+G	0.2636	0.1938	0.2670	0.2757	3.4753	4.1031	1.4825	0.7325	7.4200	1.0000	0.0000	0.4565
Con-catenated	GTR+I+G	0.2938	0.2298	0.2374	0.2391	0.9792	2.1595	0.3232	0.3399	5.6018	1.0000	0.3961	0.2101
