# Supplementary figures and images for: Multilocus sequence analysis of Treponema denticola strains of diverse origin
Source: BMC Microbiol. 2013 Feb 4;13:24. doi: 10.1186/1471-2180-13-24 (PMC3574001; doi:10.1186/1471-2180-13-24)

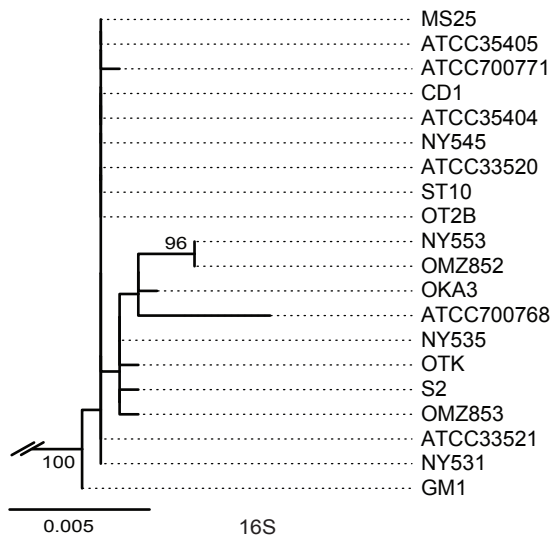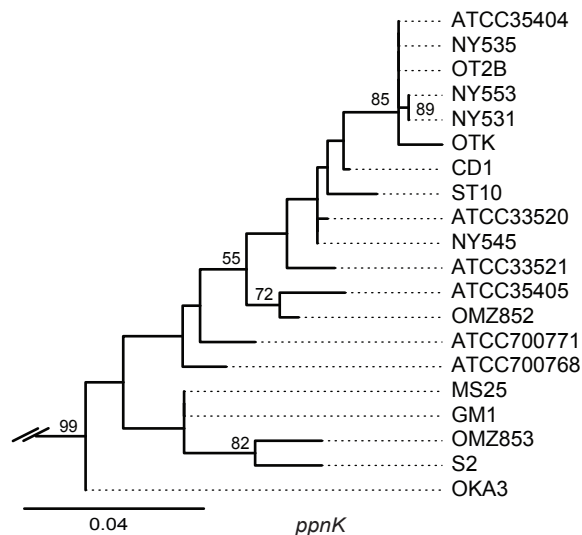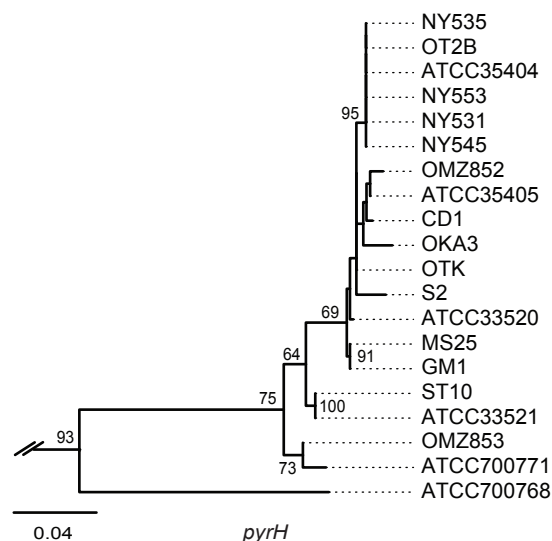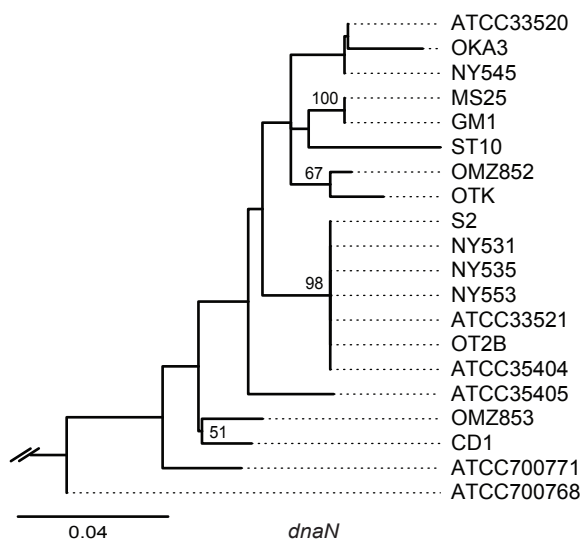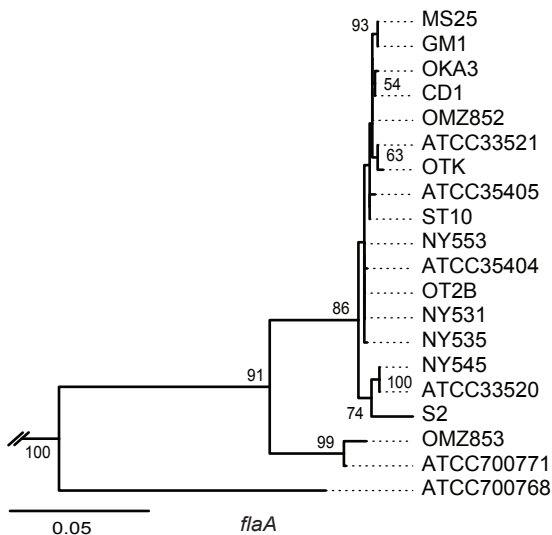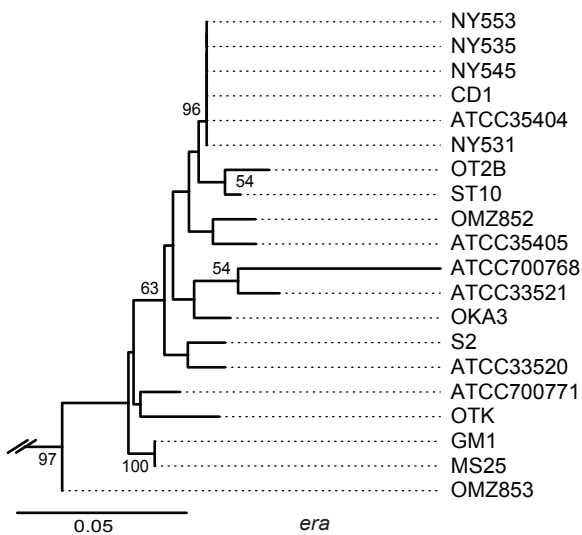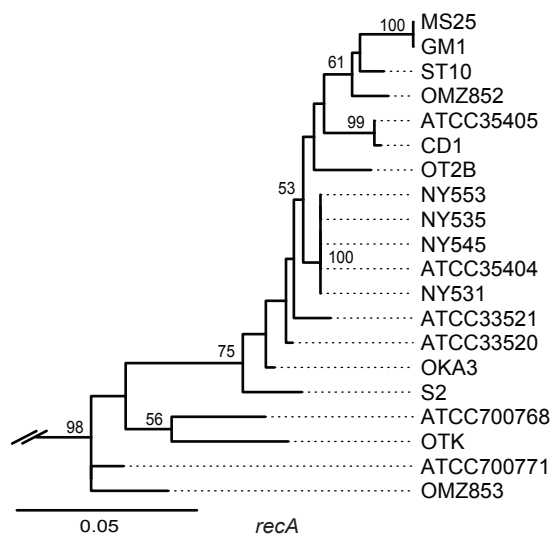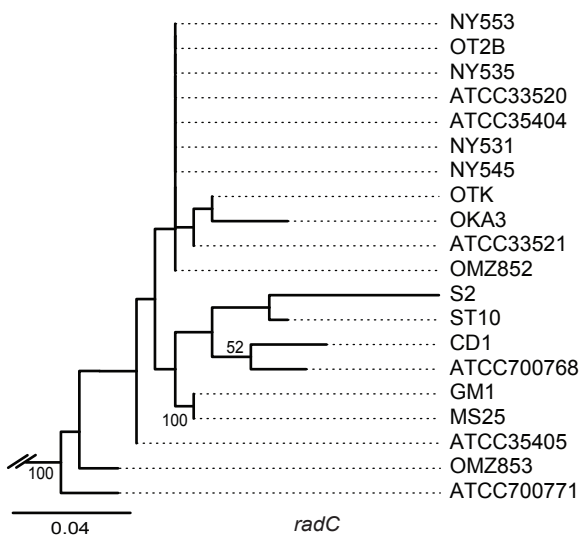

Supplement: Additional file 4 — Maximum likelihood (ML) phylogenetic trees obtained for the individual 16S rRNA, flaA, recA, pyrH, ppnK, dnaN, era and radC gene datasets. [file 1471-2180-13-24-S4.pdf]
